# Autonomic Nervous System Responses During Perception of Masked Speech may Reflect Constructs other than Subjective Listening Effort

**DOI:** 10.3389/fpsyg.2016.00263

**Published:** 2016-03-01

**Authors:** Alexander L. Francis, Megan K. MacPherson, Bharath Chandrasekaran, Ann M. Alvar

**Affiliations:** ^1^Department of Speech, Language and Hearing Sciences, Purdue University, West LafayetteIN, USA; ^2^School of Communication Science and Disorders, Florida State University, TallahasseeFL, USA; ^3^Department of Communication Sciences and Disorders, University of Texas at AustinAustin, TX, USA

**Keywords:** listening effort, psychophysiology, informational masking

## Abstract

Typically, understanding speech seems effortless and automatic. However, a variety of factors may, independently or interactively, make listening more effortful. Physiological measures may help to distinguish between the application of different cognitive mechanisms whose operation is perceived as effortful. In the present study, physiological and behavioral measures associated with task demand were collected along with behavioral measures of performance while participants listened to and repeated sentences. The goal was to measure psychophysiological reactivity associated with three degraded listening conditions, each of which differed in terms of the source of the difficulty (distortion, energetic masking, and informational masking), and therefore were expected to engage different cognitive mechanisms. These conditions were chosen to be matched for overall performance (keywords correct), and were compared to listening to unmasked speech produced by a natural voice. The three degraded conditions were: (1) Unmasked speech produced by a computer speech synthesizer, (2) Speech produced by a natural voice and masked byspeech-shaped noise and (3) Speech produced by a natural voice and masked by two-talker babble. Masked conditions were both presented at a -8 dB signal to noise ratio (SNR), a level shown in previous research to result in comparable levels of performance for these stimuli and maskers. Performance was measured in terms of proportion of key words identified correctly, and task demand or effort was quantified subjectively by self-report. Measures of psychophysiological reactivity included electrodermal (skin conductance) response frequency and amplitude, blood pulse amplitude and pulse rate. Results suggest that the two masked conditions evoked stronger psychophysiological reactivity than did the two unmasked conditions even when behavioral measures of listening performance and listeners’ subjective perception of task demand were comparable across the three degraded conditions.

## Introduction

In the normal case, understanding speech may seem to be effortless and automatic. However, even small changes in hearing acuity, signal quality or listening context can substantially reduce recognition performance and subsequent understanding or recall of the message (Van Engen et al., 2012) and presumably therefore increase perceived listening effort. Chronic effortful listening may, in turn, lead to long-term stress and fatigue as well as potentially serious health issues including hypertension and increased risk of stroke (Hogan et al., 2009). In the audiology clinic, listening effort is increasingly being seen as a significant factor for hearing aid users, both as it relates to intelligibility and as a potentially independent quality associated with willingness to adopt and continue using hearing aids (Picou, 2013). Listening effort is often associated with the allocation of limited supplies of cognitive “resources” such as working memory capacity or selective attention (Hicks and Tharpe, 2002), such that increased listening effort is associated with poorer performance on simultaneous or immediately subsequent cognitively demanding tasks (McCoy et al., 2005; Sarampalis et al., 2009). However, there is still a great deal of disagreement regarding the source of listening effort, or how to best characterize and quantify it (McGarrigle et al., 2014). The present article addresses these questions by quantifying psychophysiological responses to stimulus manipulations that are associated with different possible sources of increased listening effort.

According to one prominent proposal, the *effortfulness hypothesis*, the increase in perceived effort (and the decrease in downstream task performance) that is associated with listening in adverse conditions is linked to the acoustic phonetic degradation of the signal. Listeners confronted with a phonetically ambiguous or misleading acoustic signal must engage cognitively demanding mechanisms of repair or compensation in order to successfully decipher the intended message. Operating these mechanisms is assumed to require the commitment of cognitive resources that are in limited supply, and the consumption of these resources is typically associated with the concept of “effort.” Thus, for present purposes, effortful processes may be thought of as those cognitive processes that involve the active commitment of cognitive resources such as working memory. Because such resources are in limited supply, listeners will have fewer resources remaining for subsequent processing of the linguistic information encoded in that signal (Rabbitt, 1968, 1991; Pichora-Fuller et al., 1995; McCoy et al., 2005; Wingfield et al., 2005; Pichora-Fuller and Singh, 2006; Surprenant, 2007; Lunner et al., 2009; Tun et al., 2009). However, not all sources of signal degradation have the same effect on the signal, and it is possible that different repair or compensation mechanisms may be engaged (or the same mechanisms may be engaged to differing degrees) to achieve the same level of performance under different circumstances. That is, different types of signal degradation may incur different demands on cognitive resources, or demands on different resources, and thus may differentially affect perceived effort even when performance is comparable. The goal of the present study was to investigate this possibility by quantifying physiological responses associated with task demand while listening to three similarly intelligible but differently degraded speech signals. If different types of degradation that result in the same performance are nevertheless associated with different patterns of psychophysiological reactivity, this would suggest that listeners are engaging different compensatory cognitive mechanisms to cope with the different sources of degradation.

Three types of degradation were chosen to represent three different ways in which a signal might be degraded. The first two involve masking, and represent examples of *energetic* and *informational* masking, respectively, while the third, computer speech synthesis, represents a complex form of signal degradation accomplished without masking.

Energetic masking is the simplest type of masking, in which one signal (the masker) physically obscures some part of the meaningful (target) signal. The source of difficulty in this case is simply the physical interaction between the two competing signals in the auditory periphery (Brungart et al., 2006). Adding speech-shaped noise to the target signal is a prototypical example of energetic masking, as the decrease in performance with respect to unmasked speech is arguably due entirely to the overlap of the excitation patterns of the target and masker signals on the basilar membrane. From a listener’s perspective, the difficulty in understanding speech in noise arises mainly from the loss of information contained within those parts of the target signal that are obscured by the noise. Although listeners are likely to recognize that there are two separate sound sources in the combined signal, namely the target speech and the masking noise, they generally have little difficulty distinguishing between the two, meaning that demands on selective attention should play a minimal role in this condition (Shinn-Cunningham and Best, 2008). Similarly, the noise signal has no informational content, and therefore, in itself, is assumed to add no appreciable load to listeners’ working memory (though cf. Sörqvist and Rönnberg, 2014, who suggest that attention, and hence working memory, is still involved even in simple noise-masking conditions). In principle, the effortfulness hypothesis would thus account for any increase in listening effort related to added noise as primarily due to the need to cope with the less informative (degraded) target signal itself.

Informational masking, in contrast, is often used as a catch-all term covering all cases of interference that cannot be explained purely in terms of energetic masking (Cooke et al., 2008). In the present case we will consider a specific type of informational masking, namely the use of one or more to-be-ignored speech signals (speech maskers) to interfere with listeners’ understanding of a target speech signal, a condition under which performance has been shown to dissociate from performance under energetic masking (Brungart, 2001; Brungart et al., 2006; Van Engen et al., 2012). In this case, in addition to the energetic masking that occurs when the masking signal(s) interfere acoustically with the target signal, there is also some interference occurring at a more linguistic or cognitive level of processing (Mattys et al., 2009). For example, speech masked by two-talker babble not only presents listeners with the challenge of dealing with a partially obscured target speech signal, it also imposes greater demands on selective attention as listeners must choose to which of the three voices to attend (Freyman et al., 2004; Brungart et al., 2006; Ihlefeld and Shinn-Cunningham, 2008; Shinn-Cunningham, 2008). In addition, demands on working memory likely increase, as listeners probably retain some of the content of the masking signal in working memory and this must subsequently be selectively inhibited at the lexical level (Tun et al., 2002; Van Engen and Bradlow, 2007; Cooke et al., 2008; Mattys et al., 2009; Dekerle et al., 2014). Neuropsychological and genetic studies further suggest that populations that are predisposed to show poorer selective attention, as indexed either by increased degree of depressive symptoms (Chandrasekaran et al., 2015) or genetic markers associated with poorer executive function (Xie et al., 2015) experience greater interference in conditions that emphasize informational masking as compared to those involving primarily energetic masking.

Finally, synthetic speech represents a different sort of signal degradation, one that has been less well-studied in the effort literature but that has been shown to introduce cognitive demands on speech perception (Pisoni et al., 1985; Francis and Nusbaum, 2009). Unmasked synthetic speech, like foreign accented, dysarthric, and noise-vocoded speech consists of a single signal, thus eliminating issues of selective attention at the signal level. However, synthetic speech is distorted in ways that not only represent a lack of information, but potentially introduce misleading information (Francis et al., 2007), a property shared with accented and dysarthric speech, but not necessarily vocoded speech. Thus, listening to synthetic speech, like listening in competing speech, may require the application of additional cognitive resources for the retention and eventual inhibition of a larger number of competing lexical items in working memory (Francis and Nusbaum, 2009); but, unlike competing speech conditions, in the case of synthetic speech there is no benefit to applying selective attentional processes to filter out competing signals before their content interferes.

Thus, these three types of degradation allow for the possibility of distinguishing between listening effort due to the increased cognitive demands associated with informational masking (noise- vs. speech-masked), and of listening to a single challenging signal as compared to selectively attending to multiple signals (speech-masked vs. synthetic speech).

In order to quantify listening effort, three general methods of assessment have been identified in the literature: subjective (self-report) measures of task demand using instruments such as the NASA Task Load Index (TLX, Hart and Staveland, 1988) and the Speech, Spatial and Qualities of Hearing Scale (SSQ, Gatehouse and Noble, 2004); measures of behavioral interference between dual tasks (Sarampalis et al., 2009; Fraser et al., 2010); and physiological assessments of central nervous system function using fMRI (Wild et al., 2012) and EEG/ERP methods (Bernarding et al., 2012) and of autonomic nervous system arousal based on measurements of a variety of systems, such as those that reflect pupillary (Zekveld et al., 2011), electrodermal, and cardiovascular function (Mackersie and Cones, 2011; Mackersie et al., 2015; Seeman and Sims, 2015).

The autonomic nervous system is a division of the nervous system controlling functions vital to survival including respiration, digestion, body temperature, blood pressure, vasoconstriction, heart rate and sweating (Hamill et al., 2012). It is divided into three major branches: the *sympathetic*, *parasympathetic*, and *enteric* nervous systems. The enteric nervous system primarily governs digestion and will not be further discussed here. The sympathetic nervous system (SNS) is typically associated with *fight-or-flight* responses such as the cool, damp palms associated with confronting a physical or emotional threat, while the parasympathetic nervous system (PNS) is typically associated with rest, relaxation, and recovery from stressors. The sympathetic and parasympathetic branches interact to preserve a homeodynamic balance within the body, maintaining a stable internal state and adjusting bodily functions to respond to internal and external stimuli (Kim and Kim, 2012).

Thus, physiological measures of autonomic nervous system reactivity were selected for the present study because such measures, especially those reflecting SNS arousal, are associated both with increased cognitive demand and with emotional stress, and may therefore constitute an important link between the momentary demands of listening to speech under adverse conditions and long-term health issues associated with hearing impairment. For example, chronic stress associated with living in a noisy environment has been linked to both higher levels of SNS arousal and increased risk of adverse health outcomes (Babisch, 2011). Similarly, measures of peripheral vasoconstriction due to SNS arousal are associated with subjective measures of annoyance by noise (Conrad, 1973) which, in turn, may be among the better predictors of compliance in hearing aid users (Nabelek et al., 2006; though cf. Olsen and Brännström, 2014). Moreover, anxiety also affects speech perception, potentially increasing demand on cognitive processing (Mattys et al., 2013). Thus, developing a better understanding of autonomic nervous system responses to different sources of listening effort will also provide insight into the possibility that chronically heightened listening effort may contribute to broader issues of health and wellbeing. In this study, four measures of autonomic nervous system reactivity were assessed: skin conductance response (SCR) rate and amplitude, fingertip pulse amplitude (PA), and pulse rate (PR).

### Skin Conductance Response

The SCR refers to a phasic increase in the conductivity of the surface of the skin, especially on the palms of the hands or the feet, reflecting increased eccrine sweat gland activity. The eccrine sweat glands are innervated solely by the SNS. Skin conductance is collected by running a slight (0.5 V) current between two electrodes across the surface of the skin. As eccrine sweat gland activity increases, the concentration of negative ions on the skin surface increases, increasing conductivity between the two electrodes (Boucsein, 2012). Although SCRs are not elicited in all trials (Andreassi, 2007), their frequency and amplitude have long been associated with a wide range of psychological responses. The simplest of these is the orienting response (OR), an involuntary response to any sufficiently large change in the sensory environment, reflecting stimulus novelty and degree of surprise, but also affected by stimulus significance. In this context, the SCR is also potentiated by the arousing quality of the stimulus content (irrespective of positive or negative affective valance), such that more significant or more emotionally arousing stimuli induce a stronger SCR (Bradley, 2009). For example, Mackersie and Cones (2011) showed that increasing task demands on selective attention by increasing the complexity of a dichotic digits repetition task increased the amplitude of the SCR, suggesting that as the listening task became more attentionally demanding, listeners’ SNS arousal increased.

### Pulse Amplitude

Fingertip pulse amplitude (PA) is a measure of the volume of blood in the capillary bed of the fingertip at the peak of the heartbeat. Like the SCR, it is governed purely by the sympathetic branch of the autonomic nervous system, with increasing arousal leading to peripheral vasoconstriction and therefore decreased amplitude of the blood pulse volume signal (Iani et al., 2004; Andreassi, 2007) (henceforth PA). Phasic PA has been shown to decrease in response to increasing demands of cognitive tasks such as the Stroop task (Tulen et al., 1989) and mental arithmetic (Goldstein and Edelberg, 1997), and such decrease has been linked specifically to the increased investment of mental effort in a task, such that PA decreases parametrically with increase in working memory load (Iani et al., 2004).

### Pulse Rate

Changes in heart rate have been used extensively to study arousal related to sensory and cognitive processing. The period (and thus frequency or rate) of the heart beat is governed by both sympathetic and PNSs, with acceleration primarily under the influence of the sympathetic branch (Andreassi, 2007). Phasic cardiac acceleration and deceleration (momentary increase and decrease of heart rate) are each associated with different aspects of mental demand. Deceleration within the first few heart beats following presentation of a stimulus is typically characterized as part of an automatic OR, and is often interpreted as reflecting the holding of resources in reserve to prepare for stimulus encoding and processing (Lacey and Lacey, 1980; Lang, 1994) or even as an indication of a defensive response to threatening or unpleasant information in the stimulus (Bradley, 2009). Thus, listeners anticipating the need to process more complex or perceptually demanding stimuli, or who are experiencing the stimulus as threatening or aversive, might be expected to show a greater degree of cardiac deceleration during the initial OR. That is, to the extent that cardiac deceleration constitutes a component of an automatic OR, it is not, in itself, a reflection of the operation of an effortful (i.e., controlled, resource-demanding) process but it may nevertheless be expected to occur more strongly in conditions in which the stimulus is perceived to be aversive and/or is expected to be demanding to process further. On the other hand, heart rate has also been observed to increase as a mental task becomes more difficult, for example when doing increasingly complex mental arithmetic (Jennings, 1975), and this acceleration generally persists throughout the duration of the task. Thus, different aspects of cardiac response may reflect different ways in which a given task may be perceived as effortful: deceleration may be associated with tasks that are perceived as effortful because they involves processing stimuli that are unpleasant or demanding to encode (thus incurring a stronger OR as resources are held in reserve in anticipation of the difficult stimulus), while acceleration may be associated with tasks that are perceived as effortful because they involve significant mental elaboration or active processing of information once the stimulus has been encoded (Andreassi, 2007).

### Summary

The purpose of the present study was to quantify psychophysiological responses that might reflect differences in the degree or type of effortful cognitive mechanisms listeners employ to perceive speech under three conditions of increased difficulty compared to listening to unmasked, undistorted speech. The conditions differed in terms of the source of the difficulty (energetic masking, informational masking, and distortion) but were chosen to be matched for overall performance (keywords correct). We hypothesized that, although speech recognition performance should not differ significantly across conditions, listeners would exhibit greater psychophysiological reactivity in conditions involving informational masking and distortion, because these conditions, more so than simple energetic masking, increase demands on cognitive mechanisms of working memory and attention. In fact, results suggested instead that greater psychophysiological reactivity across degradation conditions was mainly associated with conditions involving masking (whether informational or energetic) as compared to either unmasked condition.

## Materials and Methods

### Subjects

Fourteen native speakers of American English gave informed consent and participated in this study under a protocol approved by the Purdue University Human Research Protection Program. They ranged in age from 20 to 32 years (mean = 26.0). There were 11 women and 3 men and all were right-handed. All were recruited from the Purdue University community and either had at least a Bachelor’s degree level of education (13) or were currently in college. No participant reported fluency in any language other than English. All were non-smokers in good health by self-report, and none were currently taking any medications known to influence cardiovascular or electrodermal responses. All reported having minimal or no caffeine consumption on the day of testing (though cf. Barry et al., 2008). Participants were screened for anxiety and depression which may affect or be associated with autonomic nervous system function (Dieleman et al., 2010), using scales that would be suitable for both younger and elderly individuals because this study was intended as part of a larger project including geriatric participants. All participants scored within normal limits on the Geriatric Depression Scale (GDS, Yesavage et al., 1983) and the Geriatric Anxiety Inventory (GAI, Pachana et al., 2007). All exhibited auditory thresholds within age-normal limits, passing a pure tone screening test of 20 dB SPL at 250 and 500 Hz, and 25 dB SPL at 1000, 2000, 4000, and 8000 Hz. All reported normal or corrected-to-normal vision. All participants scored within normal limits on all subscales of the Cognitive Linguistic Quick Test (CLQT, Helm-Estabrooks, 2001). Basic demographic information and test results are shown in **Table 1.**

**Table 1:** Scores range from 1 to 20 where 1 = “very low” and 20 = “very high” for ratings of mental demand, effort, and frustration, and 1 = “perfect” and 20 = “failure” for performance.

Age	GDS score	GAI score	PTA (L) dB (sd)	PTA (R) dB	CLQT attention (sd)	CLQT memory	CLQT executive function	CLQT language	CLQT visuospatial Skills
26.0	0.4 (0.9) (Max of 3)	2.8 (2.6) (Max of 7)	8.4 (4.2)	8.1 (3.4)	203.9 (8.9)	175.8 (19.6)	34.7 (2.8)	35.1 (3.2)	97.8 (3.9)


*GDS, Geriatric Depression Scale (Yesavage et al., 1983); GAI, Geriatric Anxiety Index (Pachana et al., 2007); PTA, Pure Tone Average [average of pure tones at 0.25, 0.50, 1, and 2 kHz, in the left (L) and right (R) ear]; CLQT Attention normal limits (NL) between 180-215; CLQT Memory NL between 155 and 185; CLQT Executive Function NL between 24 and 40; CLQT Language NL between 29 and 37; CLQT Visuospatial Skills NL between 82 and 105. Values are presented in the form of *Mean (SD)*.*

### Apparatus and Materials

#### Testing Environment

During the speech perception task, participants were tested in a quiet room, seated comfortably approximately 1.5 m directly in front of a speaker (Hafler M5 Reference). All stimuli were played via speaker at a comfortable listening level (approximately 76 dBA measured at the location of the seated participant’s head, averaged over four test sentences). Although this overall level is higher than is typical in speech audiometry, it corresponds to a signal (speech) level of 67 dBA combined with a masking noise level 8 dB louder (necessary to achieve comparable performance across the two masked conditions). Stimulus presentation was controlled by a program written in E-Prime 2.0 (Psychology Software Tools, Inc. [E-Prime 2.0], 2012). Responses were made verbally, and were scored on-line by the experimenter.

#### Stimuli

Stimuli were selected from a database of sentences originally developed by Van Engen et al. (2014). The subset used here consisted of 80 semantically meaningful sentences based on the Basic English Lexicon sentences (Calandruccio and Smiljanic, 2012) spoken in a conversational style by a young, female native speaker of American English. Sentences always contained four key words. For example (keywords underlined) *The hungrygirlate a sandwich.* Masking stimuli were derived from a set of 30 different sentences (not in the target set) produced by eight different female native speakers of American English (not the target talker). Two talker-babble was created by concatenating sentences from two of these talkers, removing silences, and adding them together using the mix paste function in Audacity 1.2.5^1^ The speech shaped noise was generated by filtering white noise to match the long-term average spectrum of all of the masking sentences. Thus, the two-talker babble masker clearly sounded like the speech of two talkers, while the speech shaped noise masker sounded like filtered white noise. Stimuli were mixed to present the target at a challenging SNR of -8 dB, and then all stimuli were normalized to the same RMS intensity level using Praat 5.3. At this SNR, prior studies with these stimuli in our labs have shown that comparable performance is typically elicited across the two listening conditions tested here.

Synthetic speech was generated using ESpeak 1.46^2^ Espeak is a publicly available formant-style text-to-speech synthesizer that runs under Windows and Linux. Stimuli were generated by presenting a text file with one sentence per line to the synthesizer, producing a single sound file containing all sentences spaced at regular intervals. The default voice (male) and speaking rate were used because preliminary, informal testing suggested that these were sufficiently difficult to be comparable to the masked speech stimuli in terms of overall intelligibility, even though the synthetic sentences were noticeably shorter than the natural ones. The resulting wave file was segmented into separate files using Praat 5.3, and these were subsequently RMS amplitude normalized to the same level as the masked and unmasked stimuli generated with natural speech. Thus, there were two masked conditions: speech-shaped noise and two-talker babble, and two unmasked conditions: synthetic speech and natural speech.

### Design

Participants completed two sessions, with inter-session intervals averaging 6.4 days (*SD* = 5.5; ranging from later in the same day for one participant, to 16 days later for another). In the first session, participants completed the process of informed consent, provided background demographic information, and completed screening tests for hearing thresholds, anxiety, depression, and cognitive function. In the second session participants were played two sentences (not otherwise used in the experiment) in each of the four conditions, and then completed the speech perception task, which consisted of four conditions. Each condition presented one type of stimulus: unmasked natural speech, unmasked synthetic speech, natural speech masked by speech-shaped noise, or natural speech masked by two-talker babble. Conditions were presented in random order across participants.

**FIGURE 1:**
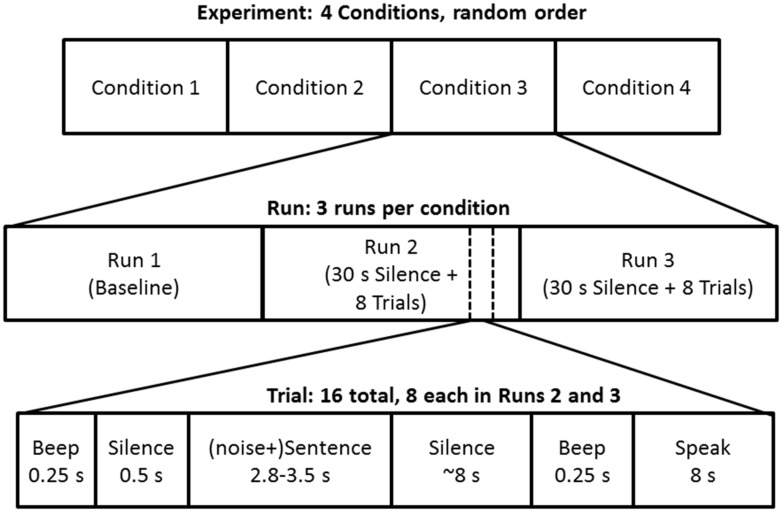
**Schematic diagram of experiment structure, highlighting the sequence of events in experimental trials within runs, and runs within conditions**.

**Figure 1** shows a schematic diagram of the experiment design. In each condition, there were three sequences of stimuli, which we will refer to here as “runs.” The first run in each condition was originally intended to permit the collection of a variety of preliminary physiological data as well as to familiarize the participant with the experimental paradigm. It consisted of 2 min of silence followed by a 0.25 s tone (400 Hz), 0.75 s of silence, 6 s of presentation of the masker (in the two masked trials) or silence in the unmasked trials. The idea was to enable the collection of data under true baseline conditions (in silence) as well as in a noise-only condition (see Parsons, 2007). This was followed by 60 s of silence and then two trials using sentences not otherwise used in the rest of the experiment. Preliminary analyses conducted after the first three participants had completed the study suggested that there was little benefit to analyzing physiological responses during the various portions of this run because some participants did not remain sufficiently still during the silent periods, so although it was included for all subsequent participants in order to maintain a consistent experimental protocol across participants, it was not further analyzed.

The second and third runs presented the experimental stimuli, and had identical formats. Each experimental run began with 30 s of silence, followed by eight experimental trials (sentences). Each trial began with a 0.25 s beep (400 Hz), followed by 0.5 s of silence, and then the start of the masking sound which began 0.75 s before the speech stimulus, resulting in a total duration of 1.5 s between the onset of the warning beep and the onset of the speech signal to be repeated. In the two unmasked conditions the period between the warning beep and the start of the speech stimulus was also 1.5 s, but the period following the beep was silent up to the beginning of the target sentence. The target sentence ended 0.25 s before the end of the noise, between 2.768 and 3.503 s after the sentence began (or 1.208–1.904 s for the synthetic speech). Twelve seconds after the initial warning beep, a second, identical beep was played to indicate to the listener that they should repeat the sentence they heard, or as much of it as they could remember. Eight seconds later the next trial began. Thus, each trial, from initial warning beep to beginning of the next trial, lasted 20.5 s while each run consisted of the presentation of eight sentences and lasted 3 min, 14 s. In total, each condition (three runs, including two containing eight sentences each) lasted 10 min, 16 s, and the entire session required a minimum of 41 min, 4 s (although exact durations varied somewhat because of different times spent between runs and between conditions). All participants finished the second session in under an hour.

### Behavioral Measures

During the speech perception task, the experimenter scored the number of key words repeated correctly on each sentence. Each sentence contained four keywords, so there were a total of 64 possible correct responses in each condition (four words per sentence, eight sentences per run, two runs per condition). The experimenter also administered an abbreviated version of the NASA Task Load Index (TLX; Hart and Staveland, 1988) after each block. Following Mackersie et al. (2015), the present study included only four of the six subscales from the orginal TLX (Mental Demand, Performance, Effort, and Frustration) and slightly revised the questions to make them more appropriae for the current listening task context. The other two dimensions, Physical Demand and Temporal Demand, were excluded in the interest of time, and because this listening task did not impose any physical or response time demand on participants. This task was administered orally, asking participants to rate each measure on a scale of 0–20, in order to permit the participant to remain still during performance of the task.

### Physiological Recordings

Immediately prior to the speech perception task period, participants washed their hands carefully with soap and water, and let them dry thoroughly. During the task, autonomic nervous system responses were collected using a Biopac MP150 Data Acquisition System, including a Biopac GSR100C amplifier (electrodermal response) and PPG100C (pulse plethysmograph) amplifiers. Acquisition and analysis was conducted using AcqKnowledge 4.3 software (Biopac Systems, Inc.) running on a Dell Latitude E6430 running Windows 7.

#### Electrodermal Response Measures

Self-adhesive Ag/AgCl electrodes for measuring skin conductance were affixed to the palmar surface of the medial phalanges of the first (index) and second (middle) finger on the participant’s right hand. Following recommended procedures, the electrodes were left in place for at least 5 min before data collection began (Potter and Bolls, 2012). The tonic conductance, in microSiemens (μS), between the two electrodes was recorded with an initial gain of 5 μΩ/V and at a sampling rate of 2.5 kHz. The signal was subsequently resampled to 19.5 Hz to facilitate digital processing (see Arnold et al., 2014 for comparable methods). The resulting tonic skin conductance level (SCL) curve was then smoothed using the built-in AcqKnowledge algorithm with baseline removal (baseline estimation window width of 1 s), and phasic SCRs were automatically identified from this signal as peaks greater than 0.01 μS occurring within a window beginning 1 s after the warning tone (to avoid including responses to the tone itself) and ending 10 s later (about 1 s before the tone indicating that participants should begin speaking). Two SCR-related measures were examined:

(a) *SCR frequency* was computed as the ratio of the number of SCR events identified within a given 16-trial block to the total number of trials within the block.
(b) *SCR amplitude* was calculated automatically by identifying the first peak (if any) in skin conductance within the 10 s window of analysis and then computing the difference between that peak value and the value of the sampling point immediately preceding the beginning of the upward inflection for that peak.

#### Blood Pulse Measures

A pulse plethysmograph transducer (TSD200) was affixed securely but comfortably using a Velcro band to the palmar surface of the distal phalange of the participant’s right ring (third) finger. This transducer emits an infrared signal and calculates the amount that has been reflected by the blood volume in the capillary bed it faces (Berntson et al., 2007). Reflectance, and thus signal level, increases with increased capillary blood volume. This signal, in volts, was initially digitized at a sampling rate of 2.5 kHz, was subsequently down-sampled to 312.5 Hz to facilitate digital analysis, and was then digitally band pass filtered (Hanning) between 0.5 and 3 Hz to remove potential artifacts. The resulting signal is periodic, with a frequency (PR) corresponding to heart rate. However, because this is a measure derived from capillary volume rather than directly from the heart signal, we will refer to it as PR rather than heart rate.

Following a combination of methods used by Potter et al. (2008) and Wise et al. (2009), PR and volume were calculated in 1 s increments over the 10 s beginning at the first warning beep for each trial, and referenced to the baseline (pre-stimulus) respective PR or volume calculated over the 2 s immediately preceding the beep for each trial.^3^ This resulted in scores centered around 1, with values greater than 1.0 indicating a heart rate acceleration or increase in PA and scores less than 1.0 indicating deceleration or decrease in PA. Two blood pulse measures were examined:

(a) *Pulse amplitude* was computed as the peak-to-trough distance for each pulse cycle within the analysis window beginning 2 s prior to the warning beep and ending 10 s after the beep, which was 2 s prior to the tone indicating that participants were to begin speaking.
(b) *Pulse rate*, which was calculated as the rate, in beats per minute, for each pulse cycle within the same time windows.

## Results

### Keyword Recognition (Intelligibility)

In order to meet the criteria for application of analysis of variance, proportion correct responses were transformed into rationalized arcsine units (RAU; Studebaker, 1985), shown in **Table 2.** This is simply a linear transformation of the results of a traditional arcsine transformation, with the goal of putting the transformed values into a range that is comparable to that of the original percentages over most of the range of values (i.e., between the “stretched” tails of the distribution). Results of a generalized linear model analysis of variance (ANOVA) with condition treated as a repeated measure showed a significant effect of condition, *F*(3,39) = 44.47, *p* < 0.001, $\eta_{p}^{2}$ = 0.77, with the unmasked, natural condition (115.5 RAU) being significantly better understood (*p* < 0.001 in all cases by Tukey HSD *post hoc* analysis) than the other three (speech-shaped noise = 93.3, two-talker babble = 91.9, and synthetic speech = 98.2, all values in RAU). There was also a significant difference between two-talker babble and synthetic speech (*p* = 0.04). However, there was no significant difference between speech-shaped noise or two-talker babble conditions, suggesting that, as intended, the two masked speech conditions were comparable in terms of intelligibility and both were significantly less intelligible than the unmasked speech.

**Table 2:** Behavioral and physiological measures obtained for each condition.

Measure	Condition			
	
	Unmasked Natural Speech	Speech-Shaped Noise Masker	Two-Talker Babble Masker	Unmasked Synthetic Speech
Proportion Correct (Raw)	0.997 (0.007)	0.903 (0.051)	0.894 (0.048)	0.931 (0.038)
Percent Correct (RAU)	115.46 (3.49)	93.26 (7.15)	91.86 (6.82)	98.17 (7.78)
Subjective Effort (Out of 20)	2.46 (2.29)	8.86 (2.67)	9.75 (4.06)	8.34 (4.17)
Pulse Rate (Ratio)	1.00 (0.04)	0.99 (0.05)	0.99 (0.05)	0.99 (0.05)
Pulse Amplitude (Ratio)	0.93 (0.14)	0.87 (0.19)	0.86 (0.17)	0.93 (0.15)
SCR Frequency (Per block of 16 trials)	0.33 (0.22)	0.35 (0.27)	0.47 (0.28)	0.43 (0.24)
SCR Amplitude (μS)	0.168 (0.133)	0.137 (0.119)	0.246 (0.274)	0.187 (0.180)


*Standard deviations shown in parentheses for all measures.*

### Subjective Task Demand (Self-Report)

Scores on the four Task Load Index questions (Mackersie et al., 2015) were relatively similar across three difficult conditions, as shown in **Table 3.**

**Table 3:** Mean scores on the NASA TLX subscales in each condition.

Measure	Condition			
	
	Unmasked Natural Speech	Speech-Shaped Noise Masker	Two-Talker Babble Masker	Unmasked Synthetic Speech
Mental Demand	2.14	10.29	11.36	9.21
Performance	2.43	8.07	8.14	7.79
Effort	2.57	10.21	11.36	9.79
Frustration	2.71	6.86	8.14	6.57


Because the different sub-scales of the NASA TLX address distinct theoretical constructs related to task load, separate analyses of variance were conducted to determine whether listeners’ subjective ratings of mental demand, performance, effort or frustration differed across the four conditions. Results showed significant main effects of condition for all four scales: Mental Demand, *F*(3,39) = 28.13, *p* < 0.001, $\eta_{p}^{2}$ = 0.68; Performance, *F*(3,39) = 10.13, *p* < 0.001, $\eta_{p}^{2}$ = 0.44; Effort, *F*(3,39) = 22.38, *p* < 0.001, $\eta_{p}^{2}$ = 0.63; Frustration, *F*(3,39) = 5.42, *p* = 0.003, $\eta_{p}^{2}$ = 0.29. The only post-hoc (Tukey HSD) pairwise comparisons between conditions that were statistically significant were those that included the unmasked, natural speech condition (*p* < 0.001) for all scales except Frustration, for which the comparison between unmasked natural speech and synthetic speech was significant only at the *p* = 0.047 level, while the comparisons of unmasked natural speech with the speech-shaped noise masking and two-talker babble masking conditions were both significant (*p* = 0.03 and *p* = 0.003, respectively). Although extremely tentative at this point, these results suggest that future research exploring task load for listening to speech masked by other speech might benefit from focusing specifically on listeners’ sense of frustration in addition to broader subjective measures of overall task load. Overall, these results suggest that, at least as far as can be determined by self-report, listeners found the degraded speech conditions to be comparatively more demanding than the unmasked natural speech, but not differently demanding compared to one another. However, it must be noted that all scores were relatively low (below 10 on a 20-point scale) suggesting that the overall task was not perceived as particularly demanding.

### Physiological Measures

Results from the four physiological measures, SCR frequency, SCR amplitude, PR, and PA, calculated for all four conditions are shown in **Table 2.** There were no significant (*p* < 0.05) (uncorrected) Pearson product-moment correlations between any of the measures within each of the four conditions, nor were there any significant correlations across conditions within any of the four measures. These scores were submitted to linear mixed model (SAS 9.3 PROC MIXED, SAS Institute Inc, 2011) ANOVA with repeated measures.^4^

#### Skin Conductance Response

A comparison of SCR frequency across the four conditions showed no significant effect of condition, *F*(3,39) = 2.03, *p* = 0.13, $\eta_{p}^{2}$ = 0.14. However, the ANOVA of SCR amplitude showed a significant effect of condition, *F*(3,36.2) = 3.02, *p* = 0.04, $\eta_{p}^{2}$ = 0.21. Note that three cells were omitted from this design because there were no SCR peaks for those subjects in those conditions. *Post hoc* (Tukey HSD) analyses showed that this effect was carried entirely by a significant difference between the two-talker babble and the speech-shaped noise conditions (p_adj_ = 0.031). This suggests that listeners showed a stronger electrodermal response when presented with speech in a background of two-talker babble as compared to a background of speech-shaped noise.

#### Pulse Rate

A graph of mean PR (calculated over 10 consecutive 1 s windows beginning at the warning beep prior to the stimulus and referenced as a proportion of the average PR calculated over the 2 s immediately preceding the beep) is shown in **Figure 1.**

Results of a linear mixed model analysis of variance (ANOVA) with two within-subjects measures (condition and time period) showed no significant effect of condition, *F*(3,39) = 1.31, *p* = 0.28, $\eta_{p}^{2}$ = 0.09, and no significant interaction, *F*(30,520) = 0.79, *p* = 0.79, $\eta_{p}^{2}$ = 0.04. However, the effect of Time Period was significant, *F*(10,520) = 4.65, *p* < 0.001, $\eta_{p}^{2}$ = 0.08. *Post hoc* (Tukey HSD) analyses show that point T + 7 (7 s after the start of the trial, roughly 6 s after the start of the sentence, and between 2 and 3 s after the end of the sentence/stimulus) exhibited a significantly lower PR when compared to all other time points except T + 6. T + 3 and T + 6 were also significantly different, but no other pairwise comparisons were significant at the *p* < 0.05 level. Thus, there appears to be a slight (but non-significant) increase in PR about 2–3 s after the beginning of the trial, approximately when we might expect the beginning of a response to the onset of the stimulus, followed by a significant decline in relative heart rate approximately when we might expect to see a response subsequent to the end of the stimulus. Note that a change of about 4%, as seen here, reflects a change of approximately 3 beats (or cycles) per minute, given an observed grand average *PR* of 74.7 beats per minute across all participants and conditions. Although this amount of change may seem small, it is relatively large compared to changes in PR seen in response to auditory stimuli in previous studies, e.g., Potter et al. (2008) (mean change < 1 BPM).

Even though the lack of a significant interaction effect does not strictly license examination of *post hoc* test results involving pairwise differences within the interaction (i.e., time point × condition), such planned comparisons may be informative in guiding the design of future research. Indeed, comparison of the lowest PRs for the speech-shaped noise, synthetic speech, and two-talker babble conditions vs. the Unmasked natural speech PR at the same time point (i.e., T + 6 for speech-shaped noise vs. T + 6 for Natural Speech, and T + 7 for synthetic speech and two-talker babble vs. T + 7 for Natural Speech) show large differences. Testing these differences using uncorrected *post hoc* comparisons^5^ and comparing the resulting p value to a threshold corrected for sequential multiple comparisons (Holm, 1979) shows that the difference for speech-shaped noise, synthetic speech and two-talker babble are all significant (*p_uncorrected_* = 0.011, 0.013, and 0.024, respectively), suggesting that degradation of speech induces significantly greater decrease in heart rate than does unmasked speech, and evidence of this increased reactivity is found approximately 6–7 s following the beginning of the stimulus.

#### Pulse Amplitude

**FIGURE 2:**
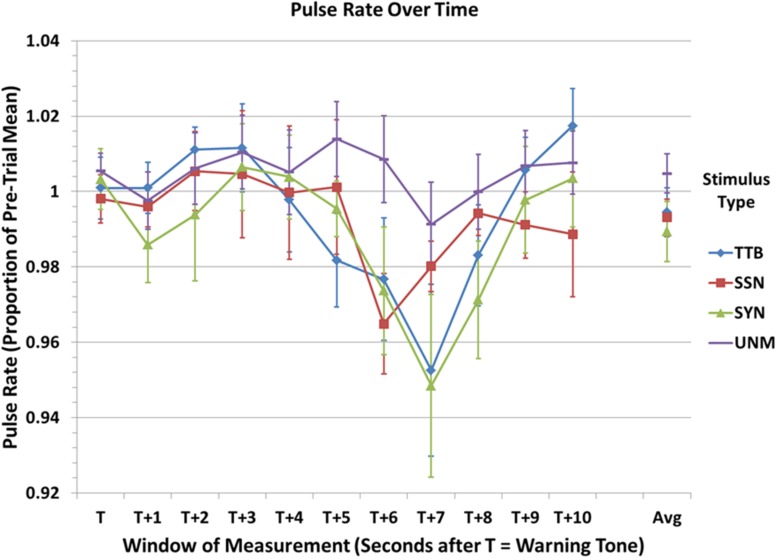
**Mean pulse rate (PR) at 1 s intervals over a 10 s window of analysis beginning at the warning beep at the start of the trial, expressed as a proportion of the mean PR calculated over the 2 s immediately preceding the onset of the trial.** TTB, natural speech masked with two-talker babble; SSN, natural speech masked with speech-shaped noise; SYN, unmasked computer synthesized speech; UNM, unmasked natural speech. Error bars indicate standard error of the mean. See text for statistical comparisons.

A graph of mean PA over 10 consecutive 1 s windows and referenced as a proportion of the average PA over the 2 s immediately preceding the beep in a manner comparable to that of PR in **Figure 1**, is shown in **Figure 2.**

Results of a linear mixed models ANOVA with two repeated measures (condition and time period) showed a significant main effect of condition, *F*(3,39) = 3.52, *p* = 0.02, $\eta_{p}^{2}$ = 0.21, and of time period, *F*(10,520) = 59.06, *p* < 0.001, $\eta_{p}^{2}$ = 0.53, but no interaction, *F*(30,520) = 0.95, *p* = 0.54, $\eta_{p}^{2}$ = 0.05. *Post hoc* (Tukey HSD) analyses show no significant pairwise differences between conditions (*p* > 0.08 in all cases). However, *post hoc* (Tukey HSD) analyses of pairwise differences in time point were found to be significant (*p* < 0.05 in all cases reported here) as follows: T vs. T + 5 and beyond; T + 1 vs. T + 4 and beyond; T + 2 vs. T + 4 and beyond; T + 3 vs. T + 5 and beyond; T + 4 vs. T + 5 and beyond; T + 5 vs. T + 6 and beyond; T + 6 and T + 7 and beyond.

Although the interaction between time-point and condition was not significant, and none of the pairwise comparisons between conditions overall or at the same time point were significant in a corrected (Tukey HSD) analysis, as with the PR date discussed above, unlicensed examination of subsidiary effects may provide guidance for subsequent research. In this spirit, examination of the graph combined with pairwise comparisons between conditions suggest that the significant effect of condition is possibly being carried by a difference between masked and unmasked conditions. According to these analyses, there does not appear to be any meaningful difference between the two masked conditions: speech-shaped noise vs. two-talker babble, *p_uncorrected_* = 0.748; Unmasked natural speech vs. synthetic speech, *p_uncorrected_* = 0.968, but there are visible differences between the two unmasked conditions that are significant by uncorrected *post hoc* analyses (although these are not significant when compared to a Bonferroni–Holm-corrected threshold): speech-shaped noise vs. unmasked natural Speech, *p_uncorrected_* = 0.042; speech-shaped noise vs. synthetic speech, *p_uncorrected_* = 0.038; two-talker babble vs. unmasked natural speech, *p_uncorrected_* = 0.020; two-talker babble vs. synthetic speech, *p_uncorrected_* = 0.018).^6^ Further, it appears that the preponderance of any such effects occurs in the last 5 or 6 time periods, a time at which the masked stimuli (speech-shaped noise and two-talker babble) exhibit considerably lower PA values than do the unmasked stimuli (Natural Speech and synthetic speech). Specifically, the greatest difference appears to be occurring around time point T + 8 or T + 9, with the divergence beginning around time T + 5 or T + 6. It may be noted that the peak PA response (at T + 9) is occurring about 2 s later than the peak PR deceleration (T + 7), though they begin at about the same time. This may be due to differences in the speed of response of the two measures or to the cognitive phenomena to which they are related, or both (see Discussion). Although these results must be considered preliminary due to the increased probability of Type 1 error through the reliance on uncorrected *post hoc* statistical analyses, overall it can be said that there appears to be a difference in the magnitude of the PA response to masked as compared to unmasked speech, and this difference begins to become apparent approximately 5–6 s after stimulus onset, and peaks 2–3 s after that.

#### Correlations

**FIGURE 3:**
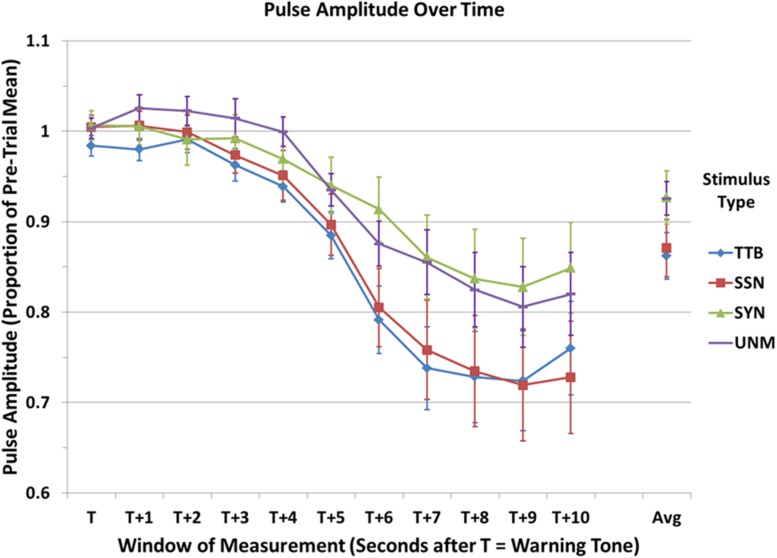
**Mean pulse amplitude (PA) at 1 s intervals over the 10 s window of analysis, expressed as a proportion of the mean PA over the 2 s immediately preceding the onset of the trial.** TTB, natural speech masked with two-talker babble; SSN, natural speech masked with speech-shaped noise; SYN, unmasked computer synthesized speech; UNM, unmasked natural speech. Error bars indicate standard error of the mean. See text for statistical comparisons.

In order to explore possible relationships between subjective measures of task demand and individual physiological responses, Pearson product-moment correlations were carried out for each of the four conditions between all four subscales of the TLX collected here (Mental Demand, Performance, Effort and Frustration) and six physiological measures: SCR Frequency, SCR Amplitude, Mean Pulse Rate, Mean Pulse Amplitude, and Pulse Rate and Amplitude at the respective minima shown in **Figures 2** and **3** (for Pulse Amplitude this was time T + 9 for all four conditions, while for Pulse Rate this was time T + 7 for all conditions except speech-shaped noise masking, for which it was T + 6). Due to the large number of comparisons, none of these tests were significant at a level corrected for multiple comparisons (*p* < 0.002). However, a general trend was observed suggesting that the measure of Mean Pulse Volume might be more likely to correlate with TLX subscales, in that it correlated with ratings of Performance (unmasked natural speech, *r* = 0.66, *p*_uncorrected_ = 0.01; synthetic speech, *r* = 0.70, *p* = 0.005), Effort (two-talker babble Masker, *r* = 0.53, *p*_uncorrected_ = 0.05; synthetic speech, *r* = 0.56, *p*_uncorrected_ = 0.04), and Frustration (unmasked natural speech, *r* = 0.69, *p*_uncorrected_ = 0.007). The only other physiological measures correlating with a TLX subscale measure with a significance at or below *p* = 0.05 were Mean Heart Rate (with Performance in the unmasked natural speech condition, *r* = 0.71, *p* = 0.005) and Pulse Amplitude at time T + 9 (with Performance in the Speech-shaped noise masking condition).

## Discussion

Behavioral measures of performance (proportion of key words repeated correctly) and subjective task demand showed that all degraded conditions were significantly less intelligible and imposed greater task demands than the unmasked natural speech condition. Additional findings also suggest that the synthetic speech condition may have been marginally less difficult, as reflected in performance, than the two-talker babble condition, and it may also have been somewhat less frustrating in comparison to unmasked natural speech than were the two masked conditions. These findings suggest that finer-grained assessments of subjective task load and behavioral performance might be informative in future research with stimuli like those used here.

In the present study, participants showed a significant increase in SCR to sentences presented in two-talker babble as compared to those presented in speech-shaped noise. Mackersie and Cones (2011) interpreted their finding that SCRs were elevated in more difficult dichotic digit task conditions as confirming that the SCR may index task demand, but Mackersie et al. (2015), who found no effect of changing SNR (and therefore presumably task demand), moderated these findings by suggesting that SCR may only be sensitive to task demand when performance is very good and/or effort is low. The present results, however, suggest a slightly different interpretation, namely that the SCR may be most indicative of the operation of selective attention. In the present experiment, performance and ratings of task demand were comparable across the two masked conditions, yet the SCR response was significantly stronger when the masker contained intelligible speech. This also highlights a significant difference between the conditions used by Mackersie and Cones (2011) and Mackersie et al. (2015): In the former, the task involved listening to streams of spoken digits presented simultaneously to each ear (i.e., speech in the presence of intelligible masking speech). In the latter, the masker consisted of a mixture of speech signals from 5 different talkers, two of which were time-reversed, making the mixture potentially much less intelligible, and perhaps closer in intelligibility to the current speech-shaped noise condition. Further research is necessary to investigate the possibility that an increase in skin conductance may correspond to the engagement of attentional mechanisms involved in separating acoustically similar streams of speech.

In contrast, physiological measures of blood PR and PA suggested the possibility that there might be some differences between one or more of the degraded conditions and the umasked natural condition. With respect to PR, the appearance of a significant deceleration approximately 5–6 s after the start of the stimulus is consistent with the expectation that the stimuli in question require some degree of mental processing. Such deceleration is consistent with the appearance of an OR indicating the holding in reserve of cognitive resources in anticipation of having to encode a perceptually demanding stimulus. The lack of any apparent increase in PR during the span of the analysis window suggests that processing these stimuli, once they are encoded, does not require significant additional mental elaboration. Notably, in-depth (but speculative) examination of the main effect of condition suggested a difference between the synthetic and unmasked natural conditions, suggesting that the OR to the synthetic stimuli was stronger (perhaps indicating an anticipation that the stimuli would be perceptually more complex) than for the unmasked natural speech. Further inspection of the data suggested that the same might be true for the other two degraded conditions as well. Even more speculatively, it is possible that there is a slight deceleration within the first 1–2 heart beats after trial onset (time T + 1) followed by a small acceleration (T + 2, T + 3) prior to the large deceleration discussed here. Such a triphasic response (deceleration, acceleration, deceleration) would be consistent with results observed from studies with shorter and less meaningful auditory stimuli (Keefe and Johnson, 1970; Graham and Slaby, 1973; cited in Andreassi, 2007 p. 354). In short, it seems likely that all three types of degraded speech required greater commitment of cognitive resources in the service of initial encoding of the signal (as indicated by a stronger OR for these stimuli), but that synthetic speech may have incurred the greatest demand. Further research is necessary to better specify the structure of the heart rate response associated with auditory stimuli of sentence length, and to better quantify factors that affect different components of this response.

Finally, there is a clear decrease in PA peaking approximately 4–5 s following the end of the stimulus. Decreased PA has long been associated with an increased demand on working memory capacity (Iani et al., 2004), so this pattern is consistent with the hypothesis that listeners were engaging working memory systems in processing the speech stimuli presented here. Other studies, however, have shown that decreased PA is a physiological response associated with the presence of noise even when task performance is unaffected (Kryter and Poza, 1980; Millar and Steels, 1990). This is then interpreted in terms of the “adaptive costs” model of physiological response to performance under stress, such that decreased PA (and other SNS responses) are considered to reflect “active coping,” that is, the application of increased effort to maintain performance in the presence of an environmental stressor (see discussion by Parsons, 2007). Indeed, research by Mattys et al. (2013) suggests that exogenously induced anxiety or stress can influence the application of capacity-demanding processes to speech perception. This interpretation would be consistent with the tentative determination that there may be a difference in the decrease in PA associated with conditions containing added noise (two-talker babble and speech-shaped noise) as compared to that associated with conditions without noise (unmasked natural speech and synthetic speech). If the reliability of this distinction is borne out by future research, its appearance here may be interpreted as reflecting either a greater commitment of working memory resources to the listening task in the two masked speech conditions as compared to the unmasked conditions, or (also) a more complex response incorporating both an autonomic stress response associated with performing a task in noise as well as the greater cognitive effort required to maintain performance when listening to degraded speech. Further research is necessary to determine whether there is in fact a reliable distinction between the PA response to speech in noise as compared to similarly difficult unmasked speech, and, if so, to further untangle direct and indirect effects of noise on the application of working memory to speech perception in both masked and unmasked conditions.

While the determination that there is an overall increased commitment of working memory capacity to speech perception in degraded conditions would be completely consistent with the predictions of the effortfulness hypothesis, the apparent discrepancy between the conclusions drawn from the different pulse measures (rate vs. amplitude) must still be considered. That is, why does the synthetic speech condition, which was significantly more difficult to understand than the unmasked natural speech condition according to both self-reported effort ratings and performance measures, seem to incur greater demand on mental processing as indexed by PA, but not according to the measure of PR? One clue to an answer to this question lies in the observation that the peak of the PA marker seems to be occurring somewhat earlier during the window of analysis than did the PR response. This temporal difference likely reflects some combination of: (1) a relative delay in the responsivity of the two systems (cardiac deceleration vs. peripheral vasoconstriction), (2) differential contribution of sympathetic arousal affecting both end organs as compared with the combined effects of parasympathetic and sympathetic systems on PR, and (3) each measure reflecting a response to different stimulus processing demands.

While it is entirely likely that the two systems respond on different timescales, the fact that they show discrepant patterns of reactivity for different sorts of stimuli is also quite consistent with the idea that the two measures reflect responses to different aspects of speech processing. In this regard, it is important to note first that previous research comparing physiological responses associated with the perception of degraded (but unmasked) speech to those associated with masked speech has already suggested that these tasks may differ in terms of the degree to which cognitive processes are applied. In particular, Zekveld et al. (2014) found that noise-vocoded speech (degradation without masking) evoked a smaller pupillary response (a measure of ANS reactivity reflecting both sympathetic and parasympathetic contributions) than did noise- and speech-masked natural speech, even when performance was matched. Moreover, regional brain activity, as measured with the BOLD response, in regions associated with speech perception and selective attention (bilateral superior- and medial-temporal gyri, and dorsal anterior cingulate cortex) changed parametrically with pupil dilation, suggesting that different types of degradation result in different degrees of demand on attentional and speech processing systems specifically related to segregating target speech from competing signals. Thus, the differences between responses to masked vs. unmasked stimuli observed here in the PA measures may reflect differences in the engagement of selective attentional mechanisms associated with segregating target from masking signals. On the other hand, the response pattern observed in the PR measures may reflect overall differences in the difficulty of encoding degraded signals as such, or perhaps even differences in the degree to which masked signals are perceived as stressful, arousing or emotionally evocative (Bradley and Lang, 2000). The fact that the one pattern (PA, related to segregation) appears later in the pulse record than the other (PR, related to orienting and preparation for stimulus encoding) even though one might arguably expect segregation to incur demand earlier in processing than encoding, may be a result of differences in the speed of response of the two systems. Further research is necessary to determine whether stimulus differences that lead to differences in PR vs. PA measures are in fact associated with differential demands on segregation vs. encoding, and, if so, whether they have similar or differing effects on downstream performance (i.e., recall or understanding of the target speech, or processing of subsequent speech), as might be predicted by the effortfulness hypothesis.

In summary, the present results suggest that listening to speech in the presence of a masking sound or sounds introduces additional, or different, processing demands beyond those associated with the simple difficulty of understanding degraded speech. From the present results it cannot be determined whether these additional demands derive from the application of additional, or different, cognitive mechanisms such as those involved in selective attention (as suggested by Zekveld et al., 2014 in explaining related findings) or whether they instead reflect aspects of an affective or emotional stress-like response to the presence of a noxious stimulus (the masker). Given that anxiety may also introduce changes in the cognitive processes applied to speech perception (Mattys et al., 2013), further research is necessary to distinguish between psychophysiological and behavioral consequences of both stress and cognitive demand on speech processing in adverse conditions.

## Author Contributions

AF and MM designed the study with input from BC and AA. BC provided the stimuli. AF and AA collected the data and conducted all analyses with input from MM and BC. AF wrote the paper, and MM, BC, and AA provided comments on drafts.

## Conflict of Interest Statement

The authors declare that the research was conducted in the absence of any commercial or financial relationships that could be construed as a potential conflict of interest.

## Footnotes

1 www.audacity.sourceforge.net.

2 http://espeak.sourceforge.net/index.html.

3 A 10 s window was used because preliminary observations suggested that some listeners were (physiologically) anticipating the signal to begin speaking that occurred 12 s after the beginning of the trial. By ending the analysis window 2 s before the signal to begin speaking, it was possible to avoid including response properties that might pertain mainly to such anticipation.

4 Effect sizes were calculated independently from F and p statistics by first estimating type III sums of squares within the PROC MIXED procedure, and then applying the methods described by Bakeman (2005).

5 Uncorrected comparisons were used because standard *post hoc* corrections take all pairwise comparisons into account, drastically increasing corrected *p*-values to compensate for comparisons that are irrelevant to the present analysis. Instead, we have chosen to report raw *p*-values along with the critical *p*-value as determined by Holm–Bonferroni sequential correction as implemented for Excel by Justin Gaetano (Gaetano, 2013) for the number of pairwise comparisons that are actually relevant to the present analyses.

6 Similar results are obtained when examining pairwise comparisons at specific time points, e.g., T + 8 and T + 9.
